# Supplementary material for: Higher levels of narrativity lead to similar patterns of posterior EEG activity across individuals
Source: Front Hum Neurosci. 2023 May 10;17:1160981. doi: 10.3389/fnhum.2023.1160981 (PMC10206039; doi:10.3389/fnhum.2023.1160981)
Supplement: Supplementary file 1 [file Data_Sheet_1.docx]

## Supplementary Figures

##
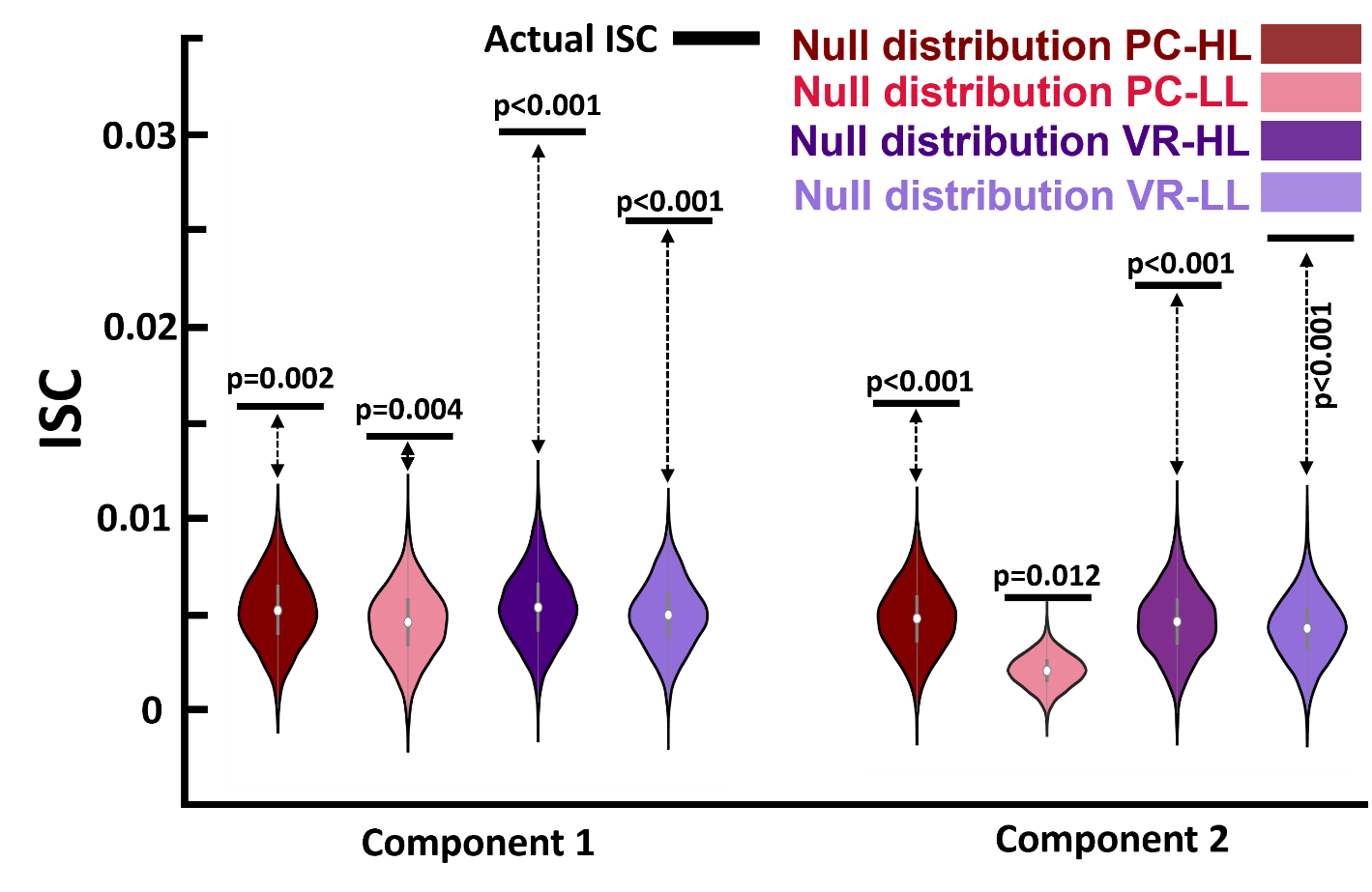


**Supplementary Figure 1 (S1):** The actual ISC against null distribution obtained from 5000 iterations of phase randomized ISC calculation. The violin plots illustrate the null distributions. The violin plots on the left side show the null distribution of component 1 (separately for four conditions, as indicated in the figure legend), and the violin plots on the right side show the same for component 2. The black lines indicate the actual values of ISC calculated from the actual EEG signals (not phase randomized). The two-sided dashed arrows indicate the distance between the null distribution and the actual value for each condition. The p-values reported above each violin plot were calculated using p = (1 + number of null ISC values ≥ empirical ISC) / (1 + number of permutations). The figure shows that in all conditions, the actual ISC is significantly above the chance level.


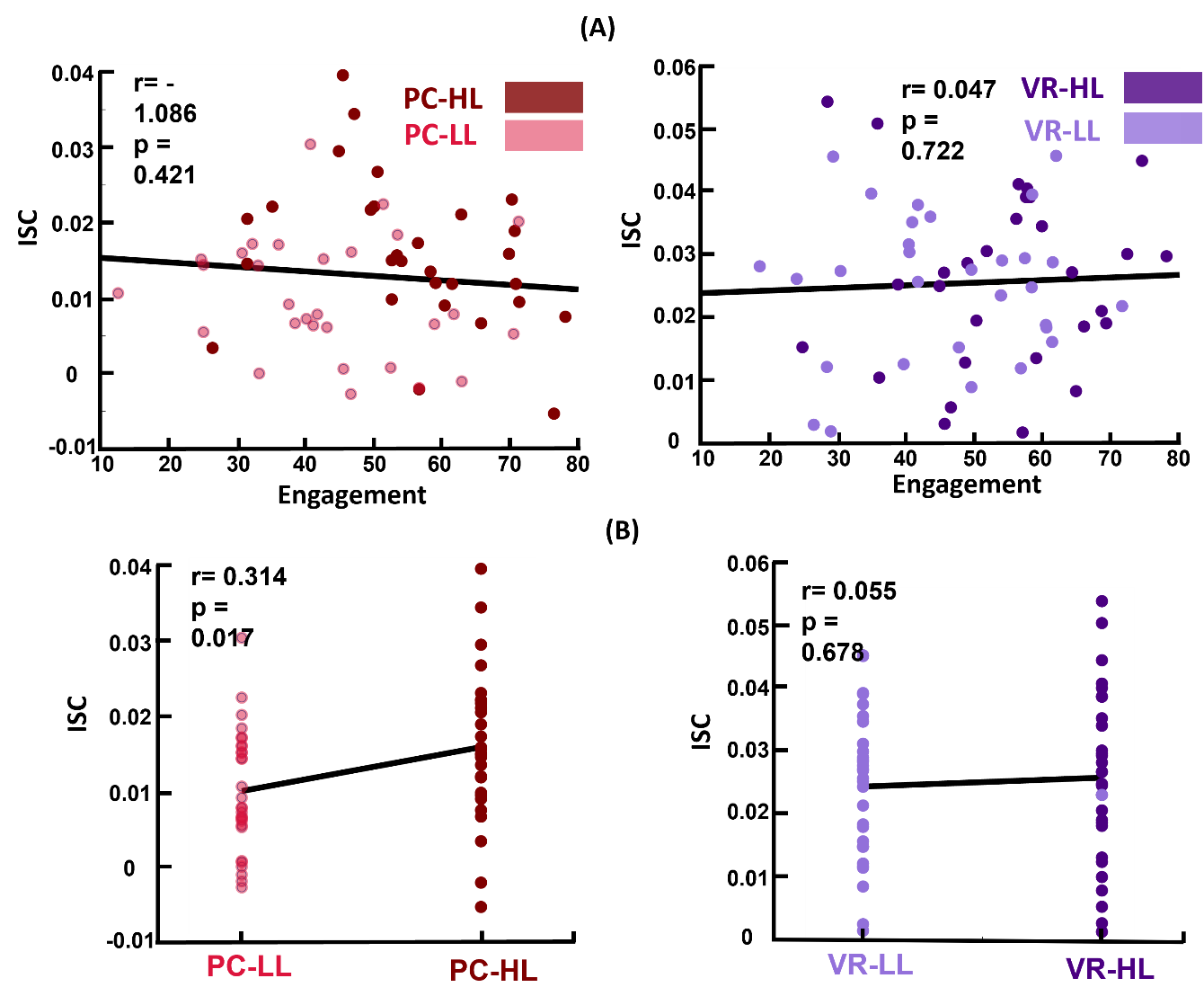


**Supplementary Figure 2 (S2):** Results of the correlation analyses for the variables of interest. The dots in the figures are according to the legends shown in panel A. The black lines show the estimate of the correlation, according to the calculated r value. The r values and the corrected p values are reported at the top of each panel. A) illustrates the correlation between ISC and engagement; the left panel refers to the PC condition and the right panel refers to the VR condition. B) illustrates the correlation between ISC and narrativity level; the left panel refers to the PC condition and the right panel refers to the VR condition.
